# Barriers and facilitators to early mobilisation and weight-bearing as tolerated after hip fracture surgery among older adults in Saudi Arabia: a qualitative study

**DOI:** 10.1093/ageing/afae075

**Published:** 2024-04-14

**Authors:** Ruqayyah Y Turabi, Katie J Sheehan, Stefanny Guerra, Matthew D L O'Connell, David Wyatt

**Affiliations:** Department of Population Health Sciences, School of Life Course and Population Sciences, King's College London, London, UK; Department of Physical Therapy, Applied Medical Sciences, Jazan University, Jazan, Saudi Arabia; Department of Population Health Sciences, School of Life Course and Population Sciences, King's College London, London, UK; Bone and Joint Health, Blizard Institute, Queen Mary University of London, London, UK; Department of Population Health Sciences, School of Life Course and Population Sciences, King's College London, London, UK; Bone and Joint Health, Blizard Institute, Queen Mary University of London, London, UK; Department of Population Health Sciences, School of Life Course and Population Sciences, King's College London, London, UK; Department of Population Health Sciences, School of Life Course and Population Sciences, King's College London, London, UK

**Keywords:** neck of femur fracture, factors, rehabilitation, interview study, orthogeriatric, mobilisation timing, precautions, qualitative research, older people

## Abstract

**Objective:**

To explore the practice of prescribing and implementing early mobilisation and weight-bearing as tolerated after hip fracture surgery in older adults and identify barriers and facilitators to their implementation.

**Methods:**

Semi-structured interviews were conducted with 20 healthcare providers (10 orthopaedic surgeons and 10 physiotherapists) from Saudi Arabian government hospitals. Data were analysed using inductive thematic analysis.

**Results:**

While early mobilisation and weight-bearing as tolerated were viewed as important by most participants, they highlighted barriers to the implementation of these practices. Most participants advocated for mobility within 48 h of surgery, aligning with international guidance; however, the implementation of weight-bearing as tolerated was varied. Some participants stressed the type of surgery undertaken as a key factor in weight-bearing prescription. For others, local protocols or clinician preference was seen as most important, the latter partially influenced by where they were trained. Interdisciplinary collaboration between orthopaedic surgeons and physiotherapists was seen as a crucial part of postoperative care and weight-bearing. Patient and family member buy-in was also noted as a key factor, as fear of further injury can impact a patient’s adherence to weight-bearing prescriptions. Participants noted a lack of standardised postoperative protocols and the need for routine patient audits to better understand current practices and outcomes.

**Conclusion:**

This study contributes to national and global discussions on the prescription of early mobilisation and weight-bearing as tolerated. It highlights the necessity for a harmonised approach, incorporating standardised, evidence-based protocols with patient-specific care, robust healthcare governance and routine audits and monitoring for quality assurance and better patient outcomes.

## Key Points

- Early mobilisation and weight-bearing as tolerated improve patient outcomes after hip fracture surgery.
- In Saudi Arabia, like many areas, there are no available data regarding mobilisation timing and weight-bearing practices.
- Weight-bearing was influenced by patient factors, fracture and fixation methods, and surgeons’ experience and training.
- Lack of standardised protocols could hinder quality assurance, weaken accountability and impact postoperative care outcomes.
- A collaborative culture, clear responsibilities and senior support would improve weight-bearing practices and outcomes.

## Introduction

Hip fractures, a primary cause of disability and mortality in older adults, pose a considerable social and medical burden given their high prevalence, health impact and healthcare cost [1]. Hip fracture surgery aims to stabilise patients, reduce pain and restore early mobility [2]. Despite the advancement in surgical repairs, studies report poor outcomes for hip fracture patients, such as decreased mobility, functional outcomes and quality of life [3, 4].

Early mobilisation (the ability to sit or stand out of bed by the day after the surgery [5]) and immediate unrestricted weight-bearing (also known as weight-bearing as tolerated or full weight-bearing) [6] are recommended after hip fracture surgery [7, 8]. Early mobilisation is associated with a reduction in postoperative complications, including pressure sores, deep vein thrombosis, pneumonia, delirium and mortality [9, 10]. Weight-bearing as tolerated is associated with reduced length of stay and improved functional outcomes compared with partial or non-weight-bearing [11–13]. Despite these benefits, they are not consistently prescribed globally [14–16]. For example, a recent scoping review identified 47 barriers and facilitators to weight-bearing as tolerated, categorised into patient, process and structural factors [17]. While patient factors were most prevalent, process factors like time to surgery and implant type also influenced weight-bearing prescription.

In Saudi Arabia, the estimated number of hip fractures among people over 50 is projected to increase from 2,949 in 2015 to 20,328 by 2050 [18]. The cost of hospital admission of 7,528 hip fracture incidents in the Eastern province alone is nearly 151 million USD, with a 1-year postoperative cost of 628.95 million USD [19]. Studies reported mortality rates of 11.1 and 26.98% in the first and second postoperative year, respectively [20, 21]. Despite studies recommending a review of care for patients with hip fractures in Saudi Arabia due to high morbidity and mortality [20, 21], no data exist on postoperative mobilisation timing and weight-bearing prescription. This limits the ability of healthcare providers to implement quality improvement initiatives to improve patient outcomes.

To address the gap, this study aims to (i) gain an understanding of the current practice related to implementing early mobilisation and weight-bearing as tolerated after hip fracture surgery in the governmental healthcare system in Saudi Arabia, and (ii) determine the barriers and facilitators to the implementation of these practices.

## Methods

This study was reported according to the Consolidated criteria for Reporting Qualitative Research (COREQ) checklist [22].

### Study design

This study adopted an interpretivist approach, using qualitative semi-structured interviews with orthopaedic surgeons and physiotherapists in government hospitals in Saudi Arabia. This interpretive approach emphasises understanding the subjective meanings and experiences of healthcare providers, acknowledging the contingent nature of knowledge and reality [23]. Orthopaedic surgeons prescribe mobilisation timing and weight-bearing after hip fracture surgeries while physiotherapists deliver and monitor their implementation. This approach allowed us to capture nuanced data on the healthcare providers’ experiences and current practices as well as broader contextual factors that shape their experiences.

### Ethical approval

This study obtained ethical approval from the King’s College London Research Ethics Committee (LRS/DP-21/22-32819), as well as from the Ministry of Health in Saudi Arabia, including Jazan Health Ethics Committee (22112) and King Fahad Hospital Hofuf (H-05-HS-065).

### Sampling

Data were collected between November 2022 and April 2023 using purposive and snowball sampling. Orthopaedic surgeons and physiotherapists (minimum 1-year of experience) from government hospitals who work with patients admitted for hip fracture surgery were included. Participants were sampled from the five geographic regions—Eastern, Western, Central, Northern, and Southern as these regions exhibit variations in healthcare provision and population demographics. The sample was restricted to government hospitals, which are primarily operated by the Ministry of Health (MOH) (58%), and other Non-Ministry of Health (Non-MOH) hospitals (10%), providing healthcare services to the public [24]. Clinicians mainly working in private hospitals (32%) were not included.

### Recruitment

A recruitment advert was shared through official social media channels and groups, including the Saudi Arabian Physical Therapy Association and International Society of Orthopaedic and Traumatology members in Saudi Arabia. The advert included questions about specialty, region, work setting and whether participants had a year or more of clinical experience, excluding internships. R.T. screened potential participants based on their professional role, experience and work setting following response to the recruitment advert. Those eligible were contacted, provided written informed consent and scheduled for interviews. Participants were asked to share the project information with others interested in participating.

### Data collection

Qualitative data were collected through one-to-one, semi-structured interviews conducted by R.T. via videoconferencing (Microsoft Teams and Zoom) at participants’ preferred times. Interviews covered participants’ clinical experiences, and current postoperative mobility and weight-bearing practices, including timing and challenges faced. The interview guide (Supplementary File 1) was informed by a previously published study [15] and broad reviews of existing literature [17, 25]. The interview was piloted with two clinicians not involved in the study, and questions were refined based on their feedback. Interviews were conducted primarily in English, with participants having the option to switch to Arabic if preferred. The interviews were digitally recorded, transcribed verbatim and translated (from Arabic to English where needed) by an external transcription and translation service.

### Data analysis

This study employed an inductive thematic analysis approach to organise themes grounded in the qualitative data [26]. This approach develops themes from data without relying on pre-existing theories, making it ideal for exploring unknown topics and gaining fresh perspectives. The analysis was completed using NVivo (Version 14.23.0) [27]. R.T. coded all interviews, while four transcripts were double-coded by two authors (D.W. and S.G.), and discrepancies in coding were discussed until a consensus was reached, ensuring the reliability of the analysis. This process of double-coding and consensus-building enhances the credibility of the findings by incorporating multiple perspectives and mitigating individual biases in the interpretation of the data [22]. After conducting 20 interviews and analysing the data, a point was reached where no new themes were present in the qualitative data [28]. Themes were arranged in a coding tree and discussed with the research team.

### Reflexivity

The research question and methodology were developed through extensive discussions that integrated insider and outsider perspectives, ensuring cultural sensitivity and methodological robustness. R.T.’s background as a registered physiotherapist with some experience in Saudi Arabia’s healthcare system enriched the research with local context understanding, influencing the recruitment strategy by leveraging advertisements and referrals to capture diverse perspectives. The decision to offer interviews in English, with the option for participants to switch to Arabic, optimised participant rapport and data authenticity, facilitating deeper engagement. The diverse research team, including D.W.’s expertise in social science and qualitative research, K.S.’s specialisation in physiotherapy and hip fracture health services and S.G.’s experience with older people care and qualitative research, through their feedback and the double-coding process, helped unpick ‘taken-for-granted’ assumptions, refining data collection, providing external viewpoints and enriching the analysis. This collaborative dynamic fostered the reflexivity and interdisciplinary collaboration essential for deepening the study’s integrity, enhancing both its quality and authenticity.

## Results

The study included 20 participants, 10 orthopaedic surgeons and 10 physiotherapists (4 physiotherapists and 6 senior physiotherapists). The participants were from different regions of Saudi Arabia: five from the Central region, two from the Eastern region, two from the Northern region, four from the Western region and seven from the Southern region. They were affiliated with the MOH (10 participants) and non-MOH (10 participants) government hospitals. The participants in the study had a range of experience, varying from 4 to 40 years. Table 1 presents participant characteristics.

**Table 1:** The participants’ characteristics of the study

**Participant number**	**Gender**	**Speciality/Rank**	**Institution type**	**Region**	**Years of experience** ^a^
1	Male	Physiotherapist	Non-MOH	Central Region	4
2	Male	Senior Physiotherapist	MOH	Southern Region	12
3	Female	Senior Physiotherapist	Non-MOH	Western Region	13
4	Male	Orthopaedic Surgeon	MOH	Southern Region	18
5	Male	Senior Physiotherapist	Non-MOH	Western Region	9
6	Male	Orthopaedic Surgeon	Non-MOH	Eastern Region	5
7	Male	Orthopaedic Surgeon	Non-MOH	Central Region	40
8	Male	Orthopaedic Surgeon	MOH	Western Region	20
9	Male	Orthopaedic Surgeon	MOH	Western Region	10
10	Female	Senior Physiotherapist	Non-MOH	Central Region	9
11	Female	Physiotherapist	MOH	Southern Region	10
12	Male	Physiotherapist	Non-MOH	Eastern Region	17
13	Male	Senior Physiotherapist	Non-MOH	Southern Region	17
14	Female	Physiotherapist	MOH	Southern Region	8
15	Male	Orthopaedic Surgeon	MOH	Central Region	10
16	Female	Orthopaedic Surgeon	MOH	Southern Region	8
17	Male	Senior Physiotherapist	MOH	Central Region	15
18	Male	Orthopaedic Surgeon	MOH	Southern Region	8
19	Male	Orthopaedic Surgeon	Non-MOH	Northern Region	6
20	Male	Orthopaedic Surgeon	Non-MOH	Northern Region	9

^a^Orthopaedic surgeons’ experiences varied between those who reported the years of their entire career as clinicians and others specifying their experience since becoming orthopaedic surgeons or after taking a subspeciality.

Interviews ranged from 23 to 60 min (mean 36.05 min, standard deviation 9.2 min).

Four themes were identified from the data: decision-making in postoperative mobilisation and weight-bearing, interdisciplinary engagement and communication, managing expectations through patient and family engagement and education, and standardising protocols and resources for consistent care.

### Theme 1: decision-making in postoperative mobilisation and weight-bearing

The prescription of early mobilisation and weight-bearing as tolerated after hip fracture surgery was a complex process and viewed by most participants as a necessary part of current practice. While guidance suggests early mobilisation and weight-bearing as tolerated are best practices, there was variation in how these terms were understood by participants and implemented in clinical practice, with a far heavier emphasis on individual decision-making over standardised guidelines. This highlights a gap between clinical guidelines and the nuanced decisions clinicians make based on their professional judgement and experience, underscoring a core challenge in the application of evidence-based practice, reconciling standardised evidence with the realities of individual patient care.

Early mobilisation was understood by most participants as encouraging mobility within 48 h after surgery. What such mobility looked like, however, varied. For the majority, early mobilisation involved a sequence of in-bed mobility exercises, sitting on the edge of the bed and transferring to a chair, with the goal of progressing to mobility outside of bed by the second day of the surgery. For a few physiotherapists, in-bed mobility exercises followed from the second day of the surgery, but mobilisation outside of the bed was delayed to a later time from 4 to 7 days. This variation underscores the subjective nature of ‘early mobilisation’ highlighting potential ambiguities that could impact patient outcomes.

*Early morning, the next day I came to him with the resident and I let him sit with the use of the contralateral hip, I pull him, I let him sit at the edge of the bed and I will assess his general status, if his status will allow and I will ask the nurse to check his vital signs and he is happy to stand* (Participant 9, Orthopaedic surgeon, MOH).

*In general, we can say from four to seven days to start to mobilise the patient. . . when I say mobilise, I mean to move the patient out of the side of the bed. . . But from [. . .] the day after surgery, the patient is allowed to sit on the edge of the bed, the patient is allowed to start free exercise like ankle pump, isometric exercise, all this kind of thing they start *(Participant 13, Senior physiotherapist, non-MOH).

### Barriers to aligning prescribed mobility and weight-bearing with what is achieved

Most participants focused on factors related to the patient as barriers to achieving early mobility and weight-bearing as tolerated. However, several surgeons perceived prompt hip fracture surgery as necessary for the achievement of early mobilisation and weight-bearing as tolerated. They noted that delays are often attributed to preoperative optimisation, hospital overloads and limited availability of intensive care units. Surgeons expressed the impact of delayed surgeries leading to increased fear, decreased confidence and physiological challenges, therefore, not achieving mobility and weight-bearing goals.

*[I]f [older adults] become bedridden for two or three or more days, they lose some confidence and their muscles become lax [weak/atrophied] and they cannot get full control, if they lose demand over the skeletal system. So, when you delay [the surgery] the muscles get lax [weak/atrophied] and in order for the patients to move, they need good tone of flexors, extensors and abductors. The faster the surgery, the less the effect on the muscle during this period *(Participant 15, Orthopaedic surgeon, MOH).

Participants reported a wide range of physical factors, including co-morbidities and previous functional levels, perceived to influence early mobility and weight-bearing as tolerated goals. They expressed the importance of patients’ previous functional level in determining their ability to walk and recognising ambulation challenges for bed-bound or chair-bound patients. Moreover, the patient’s recovery aspirations, rooted in their previous mobility levels, were perceived as key determinants of mobilisation outcomes.

*Another patient is walking, even at seventy, he is active, he has a farm, he does full praying, he kneels, he does everything, and this patient is eager to return back to activities and he is asking me, when will you allow me to walk doctor. This kind of patient can walk from the second day* (Participant 15, Orthopaedic surgeon, MOH).

Moreover, participants noted that psychological factors such as depression, fear of falling and pain impact patient compliance and exercise consistency, potentially hindering early mobilisation and weight-bearing as tolerated. They also perceived a lack of awareness among patients and families about the benefits of these practices as a challenge to rehabilitation.

*They [older adults] are not seeing or knowing the importance of early mobilisation and the significance and how the results coming after that and the benefits coming after for the patients* (Participant 2, Senior physiotherapist, MOH).

Due to their stability, walkers and Zimmer frames were commonly prescribed for older adults after surgery. However, their efficacy was contingent on the patient’s understanding of how to correctly use them. Physiotherapists also shared instances where patients displayed emotional distress and refused to use such devices:

*Most commonly [we use] walker frames, sometimes manual assistance because some of the patients they don’t know how to use the walking frame itself. Rather than pushing down with the walking frame they pull it up, so it’s not assisting them[. I]t’s just an extra weight to them* (Participant 10, Senior physiotherapist, non-MOH).

Discharge criteria were another area of divergence between the professions. While surgeons relied predominantly on functional indicators like weight-bearing, for a physiotherapist there were perceived challenges of balancing pressure to achieve desired mobility outcomes while following regulations and protocols for early discharge. Better alignment of achievement of early mobilisation and weight-bearing as tolerated with discharge criteria could help to alleviate this perceived conflict.

Many participants perceived obesity as a barrier to achieving mobility and weight-bearing. Participants suggested that obesity is a fall risk factor and required more support, which is not always available, to prevent such falls. Furthermore, a physiotherapist also highlighted this need to protect both the patient and the professional:

*Because we need support for this old lady [who is overweight], we are concerned that she may fall down. But it is not a big concern, I mean 90% will go with weight-bearing as tolerated* (Participant 20, Orthopaedic surgeon, non-MOH).

This theme shows the context-dependent ways in which mobilisation and weight-bearing decisions are made, influenced by patient, professional and surgery-related factors. While there was a general awareness of the importance of early mobilisation and weight-bearing as tolerated, these were not uncritically enacted, but part of broader care decision-making.

### Theme 2: interdisciplinary engagement and communication

All participants viewed interdisciplinary decisions and communication as important. Most decisions appeared to be driven by surgeons. However, three physiotherapists shared their experiences communicating with surgeons to modify the weight-bearing prescription:

*Sometimes, we communicate with them [surgeons] regarding the non-weight-bearing, if the patient really cannot do this. So, we ask their permission to allow the patient to put partial weight-bearing or toe-touch weight-bearing at least, because some of them, they could not perform the non-weight-bearing* (Participant 3, Senior physiotherapist, non-MOH).

As this quotation highlights, the physiotherapist’s role is not just to enact surgeon prescriptions, but to assess the suitability of such prescriptions in real-world settings. Interdisciplinary communication here facilitates ongoing dialogue in the implementation of weight-bearing prescriptions.

The surgeon, however, is not always external to this mobilisation activity. Three surgeons mentioned their active involvement in mobility, especially the first day after surgery or during early morning rounds when physiotherapist availability may be limited.

*I will make him stand by myself with the resident. Unfortunately, at this early morning time I don’t have the physiotherapist available* (Participant 9, Orthopaedic surgeon, MOH).

The interdisciplinary approach encounters its share of challenges, as revealed through participants’ experiences. Four participants shared challenges in communication concerning disagreement or conflicts in clinical judgement concerning weight-bearing prescriptions. A surgeon and a physiotherapist expressed that conflicts are often due to the physiotherapist being hesitant to advance weight-bearing. Another physiotherapist expressed that caution was a necessity, often contradicting weight-bearing as tolerated directives in order to avoid being accountable if complications occurred. Surgeons were aware of this fear:

*Now sometimes if you write weight-bearing as tolerated, the physiotherapist will not do the weight-bearing [. . ..] because they don’t want to take that decision or that responsibility (Participant 4, Orthopaedic surgeon,* MOH).

This caution, however, did not only impact the implementation of weight-bearing as tolerated prescriptions. It also meant that if weight-bearing as tolerated was not prescribed by the surgeon, as one physiotherapist described, it would not be implemented due to concerns about what would happen if adverse events occurred following deviation from a surgeon’s prescription. This hesitation to implement a different, evidence-based prescription could suggest that while professionals may agree on early mobilisation and weight-bearing as tolerated at a theoretical level, the practice is often tempered by concerns over liability and accountability.

This theme underscores the complex interplay between surgeons and physiotherapists in postoperative mobility decisions. Although there was some communication between professionals, challenges existed in effectively sharing information and responsibilities.

### Theme 3: managing expectations through patient and family engagement and education

International guidelines advise surgery within 48 h of hip fracture, leaving less time for thorough preoperative education on postoperative rehabilitation. Some participants expressed concerns about this constraint affecting patients’ expectations after surgery and expressed that preoperative education boosts patient confidence and aids early rehabilitation.

*There is no preoperative suitable education for patients on what to expect, so usually they go for surgery, specifically those geriatric patients who go for hip fixations [. . .]and when we come on the first day after surgery or the day of the surgery and we tell them that we’re going to mobilise you and you are going to get out of bed, they get panicked* (Participant 17, Senior physiotherapist, MOH).

Participants perceived the traumatic incidence of hip fracture ignites fear avoidance behaviours. Notably, these behaviours are not only exhibited by patients, but family members, motivated by genuine concern and overprotectiveness, may also intensify these apprehensions. Early engagement with patients and their families was believed to prevent the reinforcement of negative perceptions and fear-avoidance behaviours. It sets the stage for a more receptive and proactive patient willing to collaborate in rehabilitation.

*Family bonding. . .the family wants to give the parent the maximum care that they ask them not to move, because she is in pain and once she moves she is in pain. But we overcome these problems with the discussion with the kids or children asking them to keep their mother or father to move* (Participant 20, Orthopaedic surgeon, non-MOH).

Moreover, the source of the educational message plays a crucial role in its reception. A physiotherapist (participant 11) noted: *‘From what I see with my patients, the physician word is very critical to them, and it is even more important than the physiotherapist’s word.’ *This perception highlights underlying power dynamics in healthcare settings, where advice from certain professionals, such as surgeons, may hold more weight than others, potentially affecting the success of health interventions like mobilisation and weight-bearing.

While healthcare providers and family members are influential, direct communication with patients was perceived as crucial for understanding the rationale of early mobilisation and weight-bearing as tolerated. A senior physiotherapist *(Participant 10)* shared: *‘So, explaining to [patients], telling them what’s our goals and to have the plan explained to the patient herself, not to neglect the patient, and to talk to only the family members is very important from my side’.* This perception highlights the value of making patients active partners in their care, rather than passively receiving information through family members.

This theme underscores the importance of early engagement and education for patients and families in addressing fear avoidance behaviour post-hip fracture and promoting proactive rehabilitation.

### Theme 4: standardising protocols and resources for consistent care

Participants saw structural factors as directly and indirectly influencing the implementation of early mobilisation and weight-bearing as tolerated after hip fracture surgery. Participants reported a lack of standardisation due to individual surgeon practices (even within the same institution), an absence of documented protocols and outdated physiotherapy practices. The need for policy implementation was suggested due to concerns over inconsistences and outdated practices. While some expressed a need for flexible, patient-specific protocols based on factors such as disability and pain and weight-bearing tolerance, universal guideline adoption was suggested for consistency and accountability in healthcare institutions.

*Most of the surgeons [. . .] they elected to have their own protocol, [it] may differ from surgeon to surgeon in the same hospital. So, we don’t have fixed protocol* (Participant 6, Orthopaedic surgeon, non-MOH).

*I would suggest a universal guideline, which I think, we have some of them, but the thing is, it is not being followed by all the healthcare institutes. When it comes into practice, when all the healthcare institutes follow let me say, the same protocols and everyone will be you know, accountable, responsible about following or not following [them]* (Participant 19, Orthopaedic surgeon, non-MOH).

Moreover, there are further systemic challenges that participants noted to influence patient care. These include regional healthcare disparities, marked by inconsistencies in medical aid, personnel, and monitoring of patient progress. Some surgeons expressed concerns about limited physiotherapy time due to their heavy caseloads within departments. Participants suggested providing diverse, patient-specific mobility equipment like walkers, wheelchairs, adjustable beds and lifters, which are essential for facilitating in-hospital patient mobility.

*The third thing is we need [different types of] machines to help [patients] walk, because this might not be good for the patient and need another type. Also, we need a lifting machine to lift the patients who are not able to walk* (Participant 5, Senior physiotherapist, non-MOH).

This theme highlights the perceived inconsistent postoperative protocols, underscoring the need for universal standards, policy implementation, routine patient audits and diverse mobility equipment provision for enhanced patient care.

## Discussion

This study explored orthopaedic surgeons’ and physiothera-pists’ perspectives on mobilisation and weight-bearing practices following hip fracture surgery in older adults in Saudi Arabia. Participants were overall supportive of the prescription of early mobilisation and weight-bearing as tolerated. Their perspectives highlight some of the complexity of implementing these practices in clinical settings. These complexities arise from an interplay of various factors, including patient, process, and structural factors in such decision-making and practice. Although the Theoretical Domains Framework (TDF) was not utilised as part of the initial thematic analysis, it serves as a valuable conceptual lens to deepen the discussion. TDF is a well-established framework designed to identify determinants of healthcare professionals’ behaviours in evidence-based recommendations [29]. This discussion places more focus on the ‘Professional Role and Identity’, ‘Social Influences’, ‘Environmental Context and Resources’ and ‘Knowledge’ domains, indicating how professional interactions impact practice implementation. The findings of this study contribute to a broader understanding of how clinical decision-making, clinical practice and the adoption of standardised guidelines take place in real-world settings, both in Saudi Arabia and beyond.

The study’s insights into the variability of weight-bearing prescriptions, ranging from weight-bearing as tolerated to more conservative approaches based on the type of surgery or implant, highlight the complex interplay between clinical guidelines and surgeon’s discretion. This variability is not merely a divergence from evidence-based recommendations but reflects a deeper, informed clinical judgement. Surgeons’ decisions, particularly regarding the cautious use of extramedullary fixations due to concerns about mechanical failure echoing published study [15]. These choices are predicated on an understanding of the mechanical integrity offered by different implants and the potential impact on patient recovery. Furthermore, the influence of ‘Professional Role and Identity’ is evident as surgeons navigate these decisions, balancing their professional expertise with guideline recommendations. The ‘Social Influences’ domain also emerges, with training backgrounds and geographic differences in education affecting decision-making, underscoring how external factors shape internal norms and practices. This nuanced approach to weight-bearing recommendations, where evidence, personal clinical experience and the specifics of each case converge, demonstrates the dynamic nature of applying guidelines in practice. It emphasises the need for ongoing dialogue within the medical community to reconcile these variations with the aim of optimising patient care.

Consistent with earlier research [14, 30], our findings indicate that mobilisation timing and weight-bearing prescription and achievement are influenced by patient factors (e.g. pre-fracture function), care processes (e.g. surgical approach) and structures (e.g. multidisciplinary teamwork). However, good multidisciplinary teamwork did not appear to be routine practice across participant organisations. For most physiotherapists, adherence to surgeons’ weight-bearing orders was standard, with disagreement not discussed due to concerns over accountability. Yet a minority deviated from surgeons’ directives of weight-bearing, and this deviation was not always following dialogue with the surgeon. This divergence in behaviour underscores the ‘Professional Role and Identity’ domain, where ambiguities in roles and responsibilities may hinder effective collaboration, as well as the ‘Social Influences’ domain, indicating how the dynamics within healthcare teams and the perceived need for alignment with hierarchical orders impact practices. Gaps in interprofessional communication can lead to unwarranted variation in patient outcomes [31]. Addressing these gaps extends beyond individual actions to the broader organisational context, necessitating systemic changes within healthcare institutions. This approach aligns with the ‘Environmental Context and Resources’ and ‘Social Influences’ domains, emphasising the critical role of institutional support and structure in facilitating effective interprofessional collaboration. A collaborative culture, fostered by mutual respect and clear responsibilities, and supported by senior management and leadership [32, 33], could substantially enhance patient outcomes, especially within evidence-based pathways for hip fracture patients [34].

Patient and carer education was also identified as pivotal for the successful achievement of early mobility and weight-bearing as tolerated. This educational imperative is underscored by three primary challenges that could hinder optimal patient recovery. First, ‘fear avoidance behaviours’, a psychological construct [35] commonly manifested in patients and their caregivers, could lead to sedentary practices that might undermine rehabilitation efforts. Second, a general lack of awareness about the importance of mobility and weight-bearing creates a disconnect between best practices and patient behaviours. Third, the risk of falls introduces an additional layer of complexity, making it crucial to educate patients and caregivers on preventative measures and safe mobility protocols. Given these multifaceted challenges, educational interventions must employ a robust approach that includes targeted components for fear avoidance, enhancing awareness about the importance of mobility, and offering empirically supported fall prevention strategies, directly addressing the ‘Knowledge’ domain by aiming to bridge gaps in understanding and awareness among patients and caregivers. By addressing these multifaceted concerns through a culturally sensitive, evidence-based educational approach, families and carers can be equipped with the tools and knowledge they need for successful and safe patient outcomes.

While we aimed to examine the current practices and barriers and facilitators of early mobilisation and weight-bearing as tolerated as distinct concepts, participants consistently reported overlapping factors impacting both practices during the interviews. This is important, as currently there is no national audit of hip fracture care in Saudi Arabia. Should one be implemented, the recommended minimum common dataset for audit of hip fracture care specifies an indicator for mobilisation timing but not for weight-bearing prescription [36]. From the results of the current study, it appears variation in mobilisation timing may be less problematic than that observed for weight-bearing orders. There may be a need to tease out these indicators from each other in the mindset of clinicians should a future audit be implemented in pursuit of reducing unwarranted variation.

Both our findings and previous research underscore the participants’ perception of the necessity to balance tailoring treatment protocols to individual patients with adhering to prescribed protocols [15]. These protocols should be evidence informed. However, for the current study, there was an indication of surgeon-specific protocols within the same institution. While personalised care is crucial for addressing each patient’s unique needs, for certain evidence-informed care processes, a standard organisational protocol is warranted to minimise the potential negative impact of failing to implement them as routine practice.

## Limitation

While this study benefited from the insider perspective, enriching the research with understanding and depth, it also introduced potential biases, which were mitigated by the diverse backgrounds of the research team, ensuring a critical and comprehensive examination of the data, challenging implicit assumptions and facilitating a balanced exploration of the themes. However, it has some limitations. While gaining a representative sample was neither the aim nor necessary for this qualitative study, participants were self-selecting, and this may have limited the diversity of opinions and experiences obtained in this exploratory study. Similarly, our focus on government hospitals may mean findings do not reflect experiences in private healthcare settings. Additionally, it focused on the perspectives of healthcare providers and did not include the lived experiences and views of patients, which could have provided further valuable insights into the implementation and effectiveness of mobilisation timing and weight-bearing practices. This final point, in particular, highlights the need for further research on patient experiences in this area, particularly due to the increasing need for hip fracture surgery in older adults.

## Implication and conclusion

This study sheds light on the nuanced perspectives and practices of orthopaedic surgeons and physiotherapists regarding early mobilisation and weight-bearing post-hip fracture surgery in older adults within Saudi Arabia. It contributes to local, national and global discussions on this topic by exploring barriers to the implementation of accepted clinical guidelines. Identifying these barriers highlights the need for clearer evidence-based protocols that balance personalised and standardised care. The findings advocate for clear professional roles, enhancing autonomy, and promoting interdisciplinary communication to improve healthcare efficiency, resolve conflicts and optimise outcomes. It emphasises the vital role of patient and family education, alongside the development of culturally appropriate patient education strategies, in optimising recovery outcomes. Moreover, it highlights the importance of robust healthcare governance, including routine audits and monitoring, to uphold high-quality care standards and improve patient outcomes.

Acknowledging its limitations in scope, this study calls for future research with a broader participant base to validate and expand upon these findings. Future investigations should aim to refine methodologies, test and tailor protocols to local and cultural contexts, and assess the impact of defined professional roles and patient education on postoperative care and outcomes. Studies could aim to understand patients’ barriers from patients’ perspectives and from the perspective of caregivers. Moreover, education intervention studies targeting healthcare professionals, patients and caregivers could be conducted to decrease unwarranted variations in practices. In essence, this research offers insights into improving postoperative care and outcomes for older adults following hip fracture surgery, advocating for a harmonised approach that integrates standardised, evidence-based protocols with tailored, patient-centric strategies. Continued exploration and the application of these recommendations could enhance healthcare delivery quality and efficiency for better outcomes.

## Supplementary Material

aa-23-1845-File002_afae075
