# Supplementary material for: Barriers and facilitators to early mobilisation and weight-bearing as tolerated after hip fracture surgery among older adults in Saudi Arabia: a qualitative study
Source: Age Ageing. 2024 Apr 14;53(4):afae075. doi: 10.1093/ageing/afae075 (PMC11017516; doi:10.1093/ageing/afae075)
Supplement: aa-23-1845-File002_afae075 [file aa-23-1845-file002_afae075.docx]

# Barriers and facilitators to early mobilisation and weight-bearing as tolerated after hip fracture surgery among older adults in Saudi Arabia: a qualitative study.

## Supplementary file 1: Interview guide

**Participant and institute information:**

1. Can you briefly tell us about your career to date?
2. Some hospitals follow care maps (or pathways or protocols). Does your hospital have one?

**Experiences and current practice:**

1. In your practice, when do people typically get out of bed after hip fracture surgery?
2. Why this time frame?
3. Who determines when people can get up?
4. How do they get up? (assisted/Using a hoist?)
5. The evidence promoted early mobilisation – getting out of the bed on the 1^st^ day, to what extent does this occur where you work?
6. Why do you think it doesn’t happen for all the patients?
7. What are the typical weight-bearing orders given to the patient immediately after the surgery?
8. To what extent do you encourage full weight-bearing?
9. The evidence promoted full weight bearing immediately after surgery for the majority of the patients, to what extent do you think this occur where you work?
10. Why do you think it doesn’t happen for all the patients?
11. What are your suggestions to improve this practice in clinical settings?
12. Are there any other comments you would like to make on this topic? Anything I've not asked you that you think might be relevant to our study?

**THANK YOU VERY MUCH FOR YOUR TIME**
